# Vitamin D Levels and Bone Mineral Density in Premenopausal Women Compared to Postmenopausal Women: A Multi-Centre Study From Pakistan

**DOI:** 10.7759/cureus.11439

**Published:** 2020-11-11

**Authors:** Abdul Wali Khan, Nasrullah Zadran, Abat Khan, Muhammad Ishaq, Jasvindar Kumar, Asfandyar Ibrar, Amber Tahir

**Affiliations:** 1 Internal Medicine, College of Physicians and Surgeons Pakistan, Peshawar, PAK; 2 Internal Medicine, Hayatabad Medical Complex Peshawar, Peshawar, PAK; 3 Internal Medicine, Lady Reading Hospital MTI, Peshawar, PAK; 4 Cardiology, Khyber Teaching Hospital, Peshawar, PAK; 5 Internal Medicine, Khyber Teaching Hospital, Peshawar, PAK; 6 Internal Medicine, Khyber Medical College, Peshawar, PAK; 7 Internal Medicine, Dow University of Health Sciences, Karachi, PAK

**Keywords:** bone mineral density, dexa, osteoporosis, osteomalacia, 25ohd

## Abstract

Introduction

Despite the sunny climate, women in Pakistan are prone to vitamin D deficiency and subsequent low bone mineral density. The current study explores the extent of this deficiency in both pre- and postmenopausal women in our setting.

Methodology

A cross-sectional study was conducted at the outpatient departments of Lady Reading Hospital and Hayatabad Medical Complex Peshawar, Pakistan during the time period between March 2018 and June 2019. Hundred premenopausal women (control group) and 100 postmenopausal women (study group) were inducted in the study. Serum vitamin D levels were determined in patients with suspected vitamin D deficiency. Bone mineral density (BMD) was determined for each patient and the cost of each scan was afforded by our department. A dual-energy x-ray absorptiometry (DEXA) scan was used to perform the bone mineral density assessment. The data was analyzed using Statistical Package for Social Sciences (SPSS) version 26 (IBM, Chicago, IL).

Results

Serum 25OHD concentration in postmenopausal women was significantly lower compared to premenopausal women (p<0.001). In the study group, 36.0% of women had a severe deficiency of serum vitamin D levels, whereas, in the control group, only two women suffered from severe deficiency of vitamin D. Similarly, bone mineral density was also significantly correlated with the menopausal status of the women (p<0001). It was found that three-fifths of the postmenopausal women had a low bone density. Twenty-four percent of postmenopausal women had very low BMD. In comparison, only a single premenopausal woman was found to have a Z-score of below -2.0.

Conclusion

The current study highlights the impact of menopause on vitamin D levels and BMD. In our study, we found a significant difference between vitamin D levels and BMD in women of reproductive age compared to postmenopausal women.

## Introduction

Approximately one billion people in the world suffer from vitamin D deficiency [1]. Vitamin D deficiency is a common occurrence in postmenopausal women. Out of the many sources of vitamin D, humans get most exposure through direct sunlight, a diet that should be rich in fish oil, and vitamin D or multivitamin supplements [2]. In South Asian countries like Pakistan, vitamin D deficiency seems to be a gender issue. Many women do not have access to direct sunlight due to reasons, such as those who wear the veil, stay at home all day cooking, sunblock, and also poor diet [3]. Insufficient calcium and vitamin D in the diet have led to deficiencies in postmenopausal women which may lead to increased bone loss. Vitamin D deficiency is common in postmenopausal women around the world but it is more common in Asian countries like Pakistan due to the lack of fortification of food with vitamin D, and more emphasis should be put on increasing vitamin D in the diet.

Vitamin D is an important mineral required by the body [4]. It has a very important role in the development of bones, strengthens the bones, and preventing fractures. In countries like Saudi Arabia, the government has decided to fortify food with vitamin D so that the majority of women who cover themselves or stay at home can get the daily required intake of vitamin D [5]. Insufficient vitamin D levels can also lead to diseases like osteoporosis, osteoarthritis, osteopenia due to bone loss, and increased risk of fractures. The hormone vitamin D is necessary for the absorption of the claim and also the mineralization of bones which is directly related to bone mineral density (BMD). A decrease in vitamin D will result in a decrease in BMD which will directly lead to bone loss and increase the impact of fractures. 

Bone mineral density is usually used to measure the number of inorganic minerals in our bones [6]. The area of the bone which is composed of minerals is important to maintain the strength of bone and is necessary in case of fractures or to prevent diseases like osteopenia and osteoporosis. Certain factors influence the levels of BMD including gender, age, genes, diseases that involve bone loss, alcohol consumption, sedentary lifestyle, body mass index (BMI), and low vitamin D levels. Other comorbidities such as diabetes and hypertension also have an effect on BMD [7]. After the peak bone mass is acquired between the ages of 18 years and 20 years, the skeletal maturation is completed, and BMD levels start to decline. In males, osteoporosis and low skeletal mass is not that common as it is for premenopausal and postmenopausal women leading to higher BMD in males and lower in females nearing menopause [8]. 

Moreover, previous researches have shown that there is a direct link between low vitamin D levels and BMD [9]. Bone mineral density is a good measure of diseases like osteopenia and osteoporosis. However, low bone mass can be prevented by increasing vitamin D levels in adults with the aid of fortifying foods with vitamin D, supplements, or an increased diet to get adequate values of BMD. Other factors influencing vitamin D levels and BMD were further examined in this study and need to be further researched. There is scarce local literature available highlighting the significance of vitamin D deficiency and poor BMD among women in Pakistan. Therefore, the present study aimed to evaluate the burden of vitamin D deficiency and bone mineral density in postmenopausal women in our setting.

## Materials and methods

A cross-sectional study was conducted at the outpatient departments of Lady Reading Hospital and Hayatabad Medical Complex Peshawar, Pakistan during the time period between May 2018 and May 2019. The patients presenting to the clinic with complaints of fatigue, bone pain, muscle weakness, or muscle cramps, mood changes were included in the study. Hundred premenopausal women were selected from the outpatient department and were between the ages of 35 years and 45 years. This group acted as the control. Another 100 postmenopausal women were recruited between the ages of 45 years and 75 years. Women with multiple comorbidities or those who refused to take part in the study were excluded from the study. This group was tagged as the study group. Both groups of women were included in the study after taking informed verbal and written consent. Ethical approval was obtained prior to the study. A non-probability convenient sampling technique was used to select the study participants. Proper reproductive and clinical history was recorded in a predefined proforma. The demographic characteristics, 25OHD levels, hemoglobin levels, among other laboratory values were also recorded. The participants were also inquired about any preexisting conditions such as diabetes, hypertension, osteoporosis, osteopenia, parathyroid disorders, and bone fractures. 

Serum vitamin D levels were determined for all patients with suspected vitamin D deficiency. Five milliliters of blood samples were drawn using disposable syringes through the cubital veins of the patients. The serum was separated after complete centrifugation of blood samples within two hours of collection and preserved at a temperature of 20^o^C. The serum vitamin D levels were measured using the electrochemiluminescence method. A normal level of vitamin D was defined as a serum 25OHD level of more than 20 ng/mL or 50 nmol/L. A serum vitamin D level of 12-20 ng/mL was defined as vitamin D insufficiency and a concentration lower than 12 ng/mL was regarded as vitamin D deficiency. Patients were sub-categorized according to the vitamin D category as mentioned above. Bone mineral density was determined for each participant and the cost of each scan was afforded by our department. A dual-energy x-ray absorptiometry (Hologic Delphi Dexa Scanner) scan was used to perform the bone mineral density assessment. Patients were requested to lie on a soft table after removing any metallic objects from their pockets. The scanner was then passed over the patient’s lower spine and hip. With each patient, a female chaperone was allowed; however, she remained outside the x-ray room to prevent unnecessary exposure to x-rays.

The bone density test results are interpreted using Z-scores and T-scores. A T-score depicts how dense the bone is compared to the bone density of a healthy 30-year-old adult. A T-score of -1 or higher means, the patient had normal bone density. A T-score of -1 to -2.5 indicated that the patient had low bone density or osteopenia. Whereas, a T-score of -2.5 or lower was diagnostic of osteoporosis. Patients were sub-categorized into normal density, low density (osteopenia), and lower density (osteoporosis). The Z-score is a comparison of a person's bone density with that of an average person of the same age and sex. The data was then compiled and collected using the Statistical Package for Social Sciences (SPSS) version 26 (IBM, Chicago, IL). Mean and standard deviation was used to report continuous data. Frequency and percentages were used to calculate categorical data, such as age groups (premenopausal and postmenopausal), vitamin D, and BMD categories. A chi-square test was used to find the association of serum vitamin D levels and BMD with the menopausal status of women. A p-value of less than 0.05 showed statistical insignificance.

## Results

Hundred premenopausal women (control group) and 100 postmenopausal women (study group) were inducted in the study. The mean age of women in the control and the study groups was 28.45 (7.5) years and 53.74 (8.3) years, respectively. The majority of the participants were Pushtoon, followed by Hindko speaking, Punjabi, Sindhi, and Urdu speaking. Most of the postmenopausal women suffered from diabetes mellitus and hypertension while the younger group was relatively healthier (Table 1). 

**Table 1:** Demographic characteristics of study participants BMI: body mass index; HTN: hypertension; DM: diabetes mellitus; NKCM: no known comorbidity

Demographic variables	Case (postmenopausal)	Control (premenopausal)
			Mean age (SD) in years	28.45 (7.5)	53.74 (8.3)
Ethnicity		
Pushtoon	40 (40.0%)	37 (37.0%)
Hindko speaking	21 (21.0%)	18 (18.0%)
Punjabi	14 (14.0%)	12 (12.0%)
Sindhi/Urdu speaking	10 (10.0%)	15 (15.0%)
Other	15 (15.0%)	18 (18.0%)
BMI (kg/m^2^)		
Underweight	19 (19.0%)	18 (18.0%)
Normal weight	38 (38.0%)	36 (36.0%)
Overweight	23 (23.0%)	20 (20.0%)
Obese	20 (20.0%)	26 (26.0%)
Comorbidity		
HTN	64 (64.0%)	16 (16.0%)
DM	45 (45.0%)	25 (25.0%)
NKCM	10 (10.0%)	70 (70.0%)

It was found that serum 25OHD concentration in postmenopausal women was significantly lower compared to premenopausal women (p<0.001). In the study group, 36.0% of women had a severe deficiency of serum vitamin D levels, whereas, in the control group, only two women suffered from severe deficiency of vitamin D. Similarly, BMD was also significantly correlated with the menopausal status of the women. It was found that three-fifths of the postmenopausal women had a low bone density. Twenty-four percent of postmenopausal women had a lower density. In comparison, only a single woman was found to have a lower density with a Z-score of below -2.0 (Table 2). 

**Table 2:** Association of serum 25OHD concentration and bone mineral density with the menopausal status of study participants

Variable	Postmenopausal women	Premenopausal women	p-Value
Serum 25OHD concentration			
Normal	0	34 (34.0%)	
Mild deficiency	21 (21.0%)	49 (49.0%)	<0.001
Moderate deficiency	43 (43.0%)	15 (15.0%)	
Severe deficiency	36 (36.0%)	2 (2.0%)	
Bone mineral density			
Normal density	14 (14.0%)	65 (65.0%)	
Low density	62 (62.0%)	34 (34.0%)	<0.001
Lower density	24 (24.0%)	1 (1.0%)	

## Discussion

Vitamin D and bone mineral density play a very important role in maintaining the structure and functions of the bone [10]. In our study, we found that there was a significant difference between vitamin D levels in women of reproductive age compared to postmenopausal women. There was an inverse association between age and menopausal status with the serum vitamin D levels. After reaching the age of menopause, the vitamin D reserves in the body decreases. Similarly, the bone mineral density was found to be significantly associated with the menopausal status of the women. The women in their reproductive years had significantly higher BMD compared to postmenopausal women. The majority of the postmenopausal women had a T-score of -2.5 or lower, indicating osteoporosis. This highlights the role of estrogen in maintaining the bone structure, calcium regulation, and its direct association with vitamin D levels.

A study was done in 2012 concerning the deficiency of vitamin D in women after menopause especially those who are obese and suffer from cardiovascular problems [11]. The study was done on 250 women undergoing menopause and it was found 80% of these women suffer from a deficiency of vitamin D and only 5% had optimum levels of vitamin D. Deficiency of vitamin D was recognized a major issue in this study and counseling was requested concerning lifestyle and maintaining normal BMI.

Another study was conducted concerning vitamin D and BMD in premenopausal and postmenopausal women [12]. It was found that low vitamin D levels were found in women of reproductive age and postmenopausal women with low BMD levels. Patients with vitamin D deficiency and low BMD were prescribed vitamin D and calcium supplements to prevent diseases, such as osteoporosis, osteoarthritis, and osteopenia, later in life.

A study was conducted concerning South Asian women living in the UK [13]. There was a possibility of a relationship between bone mass, vitamin D levels, and parathyroid hormone levels in women of reproductive age. Blood samples of these women were checked for PTH, albumin levels, vitamin D, calcium, and scans were done for bone mineral density was done at the lumbar spine, hip, and radius. A positive relationship was found between vitamin D and bone mineral density at the area of the spine and hip, whereas a negative relationship was found between PTH and bone mineral density at the spine and hip. It was concluded that vitamin D deficiency is very much seen in South Asian women despite awareness about the topic. A decrease in vitamin D levels automatically leads to a decrease in BMD at the wrist as well as the hip.

A review was done in 2016 on the Chinese population about the association between BMD and vitamin D levels in the blood [14]. Among Chinese people, low BMD is related to the prognosis of fractures of the hip as low vitamin D levels are associated with low BMD. A similar study was conducted in 2001 on postmenopausal women [15]. The deficiency of vitamin D was seen to increase bone loss and was a major reason for increasing the risk of fractures thereby increasing the chances of osteoporosis in healthy postmenopausal women. 

A direct relationship has been found previously, between estradiol and vitamin D levels [16]. Women with decreased vitamin D levels also have concomitantly decreased estradiol levels indicating a relationship between the two variables. In postmenopausal women, estradiol levels are reduced and hence are susceptible to vitamin D deficiency. An optimum intake of vitamin D and calcium is required to prevent bone loss [17]. The rate of loss of bone premenopause is 0.2% every year from the spinal area which rises to 0.75% postmenopause. To avoid complications with osteoporosis in postmenopausal females, they should be treated with estrogen replacement therapy or hormone replacement therapy in adjunction with increased intake of calcium supplements [17].

This study was not without its limitations. Since the study was not longitudinal, the long-term effects of vitamin D supplements were not reported. Lastly, a larger sample size with a diversified study population would have explored the sociodemographic factors related to vitamin D deficiency more thoroughly. 

Our study findings indicate that postmenopausal women had low vitamin D levels and low BMD, therefore they should be administered vitamin D supplements to prevent complications. The clinicians should encourage daily exposure to sunlight and dietary modifications. 

## Conclusions

The current study highlights the impact of menopause on vitamin D levels and BMD. In our study, we found a significant difference between vitamin D levels in women of reproductive age compared to postmenopausal women. Age was significantly associated with serum vitamin D levels. Similarly, the bone mineral density was found to be significantly associated with the menopausal status of the women. The women in their reproductive years had significantly denser BMD compared to postmenopausal women. Vitamin D and calcium supplements, daily exposure to sunlight, and dietary modifications are recommended for women with vitamin D deficiency and low BMD. 
